# Meiotic chromosomes and nucleolar behavior in testicular cells of the grassland spittlebugs *Deois flavopicta, Mahanarva fimbriolata* and *Notozulia entreriana* (Hemiptera, Auchenorrhyncha)

**DOI:** 10.1590/S1415-47572010005000025

**Published:** 2010-06-01

**Authors:** Márcia Maria Urbanin Castanhole, Luis Lênin Vicente Pereira, Hederson Vinicius de Souza, José Raul Valério, Leonardo Rodrigues Barbosa, Mary Massumi Itoyama

**Affiliations:** 1 Laboratório de Citogenética e Molecular de Insetos, Departamento de Biologia, Instituto de Biociências, Letras e Ciências Exatas, Universidade Estadual Paulista Júlio de Mesquita Filho', São José do Rio Preto, SPBrazil; 2 Empresa Brasileira de Pesquisa Agropecuária Beef Cattle, Campo Grande, MSBrazil; 3 Empresa Brasileira de Pesquisa Agropecuária Florestas, Curitiba, PRBrazil

**Keywords:** chromosomal characterization, karyotype, cytogenetics, spermatogenesis, holocentric chromosomes

## Abstract

Spittlebugs annually infest pastures and cause severe damage, representing a serious problem for the tropical American beef cattle industry. Spittlebugs are an important biotic constraint to forage production and there is a lack of cytogenetic data for this group of insects. For these reasons, we conducted this work, in which the spermatogenesis and nucleolar behavior of *Deois flavopicta*, *Mahanarva fimbriolata* and *Notozulia entreriana* were studied. The males possessed testes in the shape of a “bunch of grapes”; a variable number of testicular lobes per individual and polyploid nuclei composed of several heteropycnotic bodies. A heteropycnotic area was located in the periphery of the nucleus (prophase I); the chiasmata were terminal or interstitial; metaphases I were circular or linear and anaphase showed late migration of the sex chromosome. The chromosome complement had 2n = 19, except for *N*. *entreriana* (2n = 15); the spermatids were round with heteropycnotic material in the center and elongated with conspicuos chromatin. The analysis of testes after silver nitrate staining showed polyploid nuclei with three large and three smaller nucleolar bodies. Early prophase cells had an intensely stained nucleolar body located close to the chromatin and another less evident body located away from the chromatin. The nucleolar bodies disintegrated during diplotene. Silver staining occurred in two autosomes, in terminal and subterminal locations, the latter probably corresponding to the nucleolus organizer regions (NORs). The spermatids were round with a round nucleolar body and silver staining was observed in the medial and posterior region of the elongated part of the spermatid head.

## Introduction

The grassland spittlebugs include a composite of species belonging to the order Hemiptera, suborder Auchenorrhyncha and family Cercopidae. Insects of the suborder Auchenorrhyncha constitute important components of the entomofauna associated with forage grass. This group has approximately 1,500 species in 150 genera (Liang and Webb, 2002), mainly distributed in tropical and subtropical areas. About 400 spittlebug species are found in South America, several of which are important pests of forage grass and sugarcane fields (Costes and Webb, 2004).

*Deois*, *Mahanarva* and *Notozulia* constitute the main pests of forage grass in tropical America. The nymphs and adults of these insects can cause the death of parts of the plants. The loss of pastures attacked by these insects every year is therefore an important concern for the Brazilian beef cattle industry (Valério *et al.*, 2001).

Although spittlebugs from South and Central America represent an important group of pests, they have been relatively poorly studied. Many of these species are believed to have been described in the past because their outstanding color patterns attracted the attention of taxonomists. According to Carvalho and Webb (2004), many of these descriptions are superficial which partly explains the current shortage of information. Besides the superficial taxonomy, other aspects of these species were only partly studied, such as their bioecology and cytogenetics.

The first reported cytogenetic data on Auchenorrhyncha were those of 22 species presented by Boring (1907). Halkka (1959) described the chromosome numbers n = 5 through n = 19 and 2n = 10 through 2n = 39 for the same group. The Cercopidae species *Aphrophora forneri*, *A. alni, Neophilaenus lineatus* and *N. exclamationis*, analyzed by Halkka (1964), showed chromosome complement with 14A + XY, 14A + X0, 14A + X0 and 18A + XY, respectively. While performing cytogenetic studies of Cercopidae species of the genus *Cosmoscarta* (*C. dimidiata, C. septempunctata, C. decisa, C. elegans* and *C. fluviceps*), Dey (1991) found 2n = 28 and a XY sex-determination system, except for *C. elegans* which showed a neo-XY system. Marin-Morales *et al.* (2002) analyzed two species of Cercopidae from Brazil, *Mahanarva fimbriolata* and *M*. *posticata*, both with 2n = 19 (18A + X0).

In species in which the chromosomes display localized centromeres, the number of chiasmata may vary greatly in bivalents of different sizes. One or two chiasmata were reported in small bivalents, whereas five to eight chiasmata were observed in larger bivalents, *e.g.*, in grasshoppers (White, 1973, Jones, 1987). This observation indicates that there are no structural restrictions to chiasma formation in this type of chromosome.

Chiasma formation in holocentric chromosomes, however, follows different rules. Halkka (1964) determined the number of chiasmata in 60 auchenorrhynchan species (Homoptera, Auchenorrhyncha) belonging to six families. In all the species, bivalents displayed only one or two chiasmata. Similarly, the formation of one or two chiasmata in each bivalent seemed to be the rule in psyllid species (Homoptera, Psylloidea) (Maryanska-Nadachowska, 2002). This pattern also seems to be typical of other groups displaying holocentric chromosomes (White, 1973). Although there are a few reports of holocentric bivalents with more than two chiasmata (Tian and Yuan 1997, Kuznetsova *et al.*, 2003), the behavior of these bivalents throughout meiosis has never been traced in detail.

Therefore, there is a clear discrepancy between the importance of some cercopid species as agricultural pests in Brazil and the available cytogenetic data on these insects. In this study we analyzed the spermatogenesis, including chromosome and nucleolar behavior during meiosis and spermiogenesis, in adult males of *Deois flavopicta* (Stal, 1854), *Mahanarva fimbriolata* (Stal, 1854) and *Notozuliaentreriana* (Berg, 1879).

## Material and Methods

Fifteen specimens of the grassland spittlebugs *Deois flavopicta*, *Mahanarva fimbriolata,* and *Notozulia entreriana* were collected in *Brachiaria decumbens* pastures established on the Embrapa Beef Cattle Farm (20°27' S; 54°37' W, 530 meters) in Campo Grande, MS, Brazil. Male spittlebugs were collected while still inside the foam layer characteristically produced by the nymphs. They were therefore adults recently emerged from the foam and less than one day old. The insects were collected alive and kept inside small test tubes until being fixed in methanol:acetic acid (3:1) and stored at 4 °C. The fixed insects were dissected and their testes were removed, placed on microscope slides, stained with lacto-acetic orcein and squashed. Silver nitrate staining was performed according to Howell and Black (1980). The images were analyzed under a Zeiss AXIOSKOP 2 microscope with a 12V/100W light bulb and captured with the built-in Digital Image Processing AXIONVISION 3.1 (Zeiss) software.

## Results

The testicular cells of *Deois flavopicta*, *Mahanarva fimbriolata* and *Notozulia entreriana* were shaped like “a bunch of grapes” wrapped with a transparent membrane. The number of lobes varied among individuals: 14, 15, 17, 18, 19, 20, 22 and 25 in *D*. *flavopicta*; 32 and 34 in *M*. *fimbriolata*; and 14, 16 and 17 in *N*. *entreriana*.

**Figure 1:**
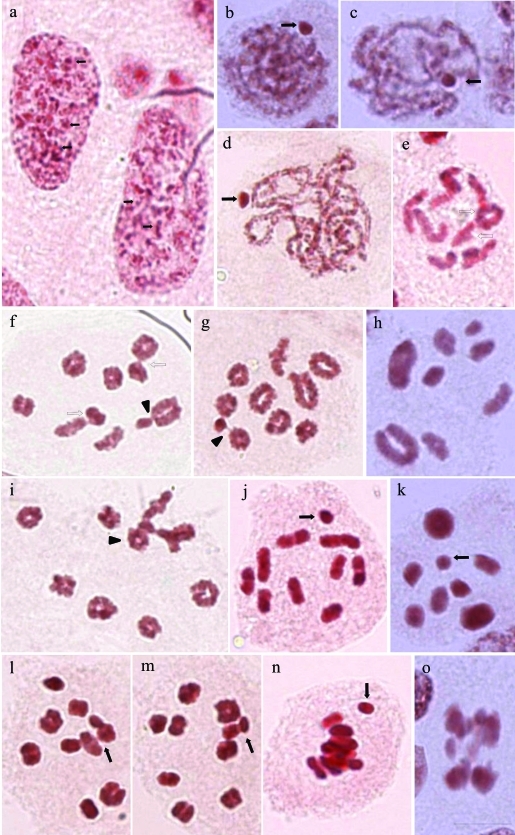
Cells of the seminiferous tubules of *Deois flavopicta* (a, e, j, n), *Mahanarva fimbriolata* (d, f, g, i, l, m), and *Notozulia entreriana* (b, c, h, k, o) stained with lacto-acetic orcein. a) Polyploid nucleus of the nutritive cells with several heteropycnotic areas of different sizes (small arrows); b-i) different stages of prophase I: leptotene (b), zygotene (c) (sex chromosome, arrows), pachytene (d) and diplotene/diakinesis (e-h) (associations between autosomal “bivalents” - hollow arrow, and association of autosomal “bivalents” and sex chromosome – arrowhead); i) cell in diplotene/diakinesis showing an association between three autosomes and the sex chromosome (arrowhead); j) metaphase I of *D*. *flavopicta* (18A + X0, X, arrow); k) polar view of a *N*. *entreriana* metaphase I with 2n = 14A + X0 (X, arrow); l, m) polar view of a *M*. *fimbriolata* metaphase I with 2n = 18A + X0 (X, arrow); n) beginning of anaphase I, with the X chromosome separated from the autosomes (arrow); o) anaphase II. Scale bar: 10 μm.

The lacto-acetic orcein-stained testes had polyploid nuclei with several evenly distributed heteropycnotic bodies of similar size (Figure 1a). In the beginning of prophase I (leptotene/zygotene), the three species showed a heteropycnotic body at the periphery of the nucleus (Figure 1b,c). This body could be seen until the end of pachytene (Figure 1d). In diplotene, the three species had autosomal bivalents (“bivalents”) with a few interstitial or terminal chiasmata (Figure 1e). In *M*. *fimbriolata* two (Figure 1f), three (Figure 1g) or four (Figure 1i,l,m) autosomal “bivalents” were observed. The sex chromosome is connected by chromatin filaments with autosomal bivalents.

Polar views of metaphases I allowed the observation that the chromosome complement of *D.flavopicta* had 2n = 18A+X0 (Figure 1j), *N*. *entreriana* presented 2n = 14A+X0 (Figure 1k) and *M*. *fimbriolata* showed 2n = 18A+X0 (Figure 1l,m). Other characteristics seen in this phase were the holocentric autosomal “bivalents” and the sex chromosomes. The small autosomal “bivalents” were of similar size and the sex chromosomes were smaller, but both had telomeric associations. At the end of metaphase I, when the chromosomes were situated in the equatorial plane of the cell (lateral view), the sex chromosomes could separate from the autosomes (Figure 1n), which was followed by the separatation of the homologous sex chromosomes and of their sister chromatids. In this case, late migration of the sex chromosomes could occur in anaphase II (Figure 1o).

**Figure 2:**
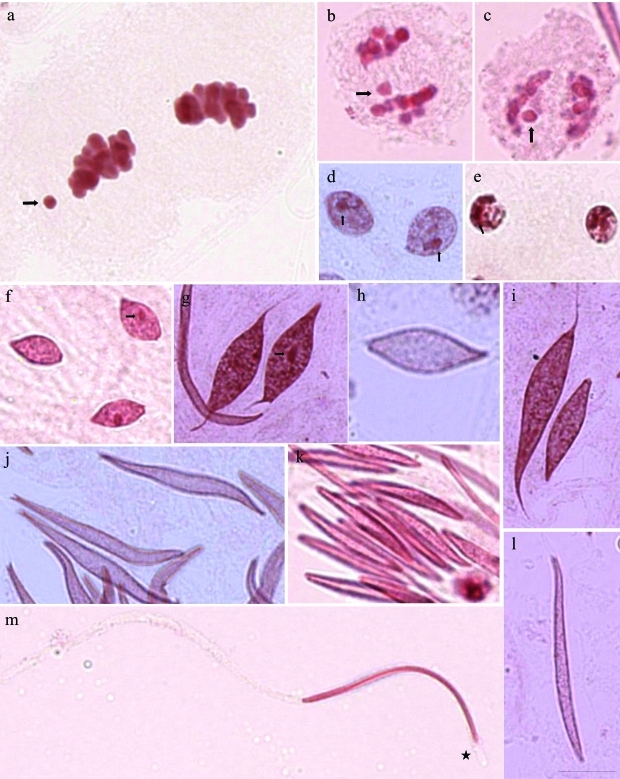
Cells of the seminiferous tubules of *Deois flavopicta* (b, c, f, k, m), *Mahanarva fimbriolata* (a, e, g, i, l) and *Notozulia entreriana* (d, h, j) stained with lacto-acetic orcein. a-c) Telophase II, the arrows indicate the sex chromosome; d) round spermatid with heterochromatic material in the center (small arrows); e) round spermatid with heterochromatin material near the nuclear envelope (small arrow); f-h) elongating spermatid with heteropycnotic material in the center in *D*. *flavopicta* (f, small arrow) and in *M.fimbriolata* (g, small arrow) and no lacto-acetic orcein staining in *N*. *entreriana* (h); the heteropycnotic material is disorganized with the elongation of the spermatid, visualized close to the envelope (i-l); (m) in the elongated spermatids, the chromatin material is in the head area, with a small space without chromatin (star). Scale bar: 10 μm.

During the first division, the autosomal “bivalents” were observed to divide reductionally, whereas the sex chromosome divided equationally. In the second meiotic division, the autosomes were equationally separated and the sex chromosome were reductionally divided. Therefore, only one daughter cell received a sex chromosome in telophase II (Figure 2a-c).

The spermiogenesis process began with round spermatids containing a heteropycnotic round area that persisted during the elongation process and was located in the spermatid periphery (Figure 2d,e). *M*. *fimbriolata* had an additional heteropycnotic material next to the nuclear envelope (Figure 2e). This heteropycnotic body was seen in the beginning of the elongating process in *D*. *flavopicta* (2f) and in *M.fimbriolata* (2g) and it dissociated in these two species, in which it stained only close to the nuclear envelope (Figure 2i-l). In *N.entreriana* the heteropycnotic body was evident close to the nuclear envelope (Figure 2h). The elongated spermatids displayed chromatin material throughout the internal area of the head, with the exception of the frontal area of the head (Figure 2m).

###  Variability in nucleolar morphology

**Figure 3:**
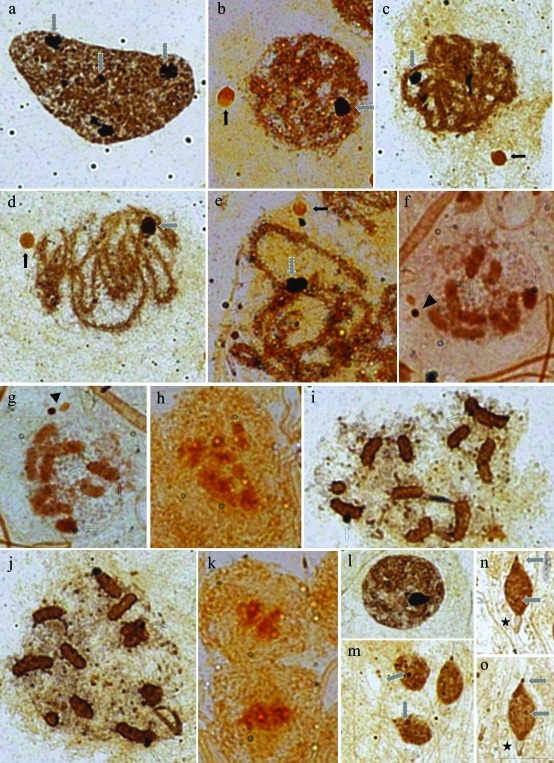
Silver stained cells of the seminiferous tubules of *Deois flavopicta* (a, c, d, i, j, l, m, n, o), *Mahanarva fimbriolata* (f, g) and *Notozulia entreriana* (b, e, h, k). a) Strongly impregnated blocks of different sizes in polyploid nuclei of the nutritive cells (dashed arrows); b,c) leptotene/zygotene with two nucleolar bodies, one more stained than the other – the darker-stained one (dashed arrow) is located in the chromatin material and the lighter-stained one (arrow) is away from the chromatin; d,e) these bodies are visible until the end of pachytene (arrows and dashed arrows), undergoing disorganization later; f,g) in *M*. *fimbriolata* metaphase I*,* the nucleolar bodies can still be observed, but have a reduced size (arrowhead). There are some impregnated regions in some autosomal “bivalents” (hollow arrows); h) in a *N*. *entreriana* metaphase I there is no silver staining; i,j) in *D. flavopica* metaphases I, silver staining is found in the periphery of the autosomal “bivalents” and in putative NOR areas (hollow arrows); k) it is not possible to see any stained material in telophase; l) round spermatid with a nucleolar body; m-o) elongating spermatids with impregnation in the posterior and medial region of the head (dashed arrows). Note an unstained area in the frontal part of the head (stars). Scale bar: 10 μm.

After silver staining, the polyploid nuclei of the nutritive cells of *Deois flavopicta*, *Mahanarva fimbriolata* and *Notozulia entreriana* showed several nucleolar bodies of variable morphology and size (Figure 3a). The cells in prophase I (leptotene-pachytene) had a strongly stained nucleolar body located close to the chromatin, and another one of the same size, but less evident, located away from the chromatin (Figure 3b-d). In zygotene/pachytene these bodies started to dissociate and could not be seen at the end of prophase I (Figure 3e). The only exception was *M*. *fimbriolata*, in which the two much smaller bodies (dark and light-stained) could be observed (Figure 3f,g). The nucleolar material in metaphase I differed among the three species: in *M*. *fimbriolata* two nucleolar bodies and some differentially stained chromosomes could be seen, which are possibly NOR-bearing (Figure 3f,g); in *N. entreriana,* these elements were not stained (Figure 3h); and in *D. flavopicta* the NORs area and the periphery of some chromosomes were stained (Figure 3i,j). There was no evidence of nucleolar material in telophase (Figure 3k).

**Figure 4:**
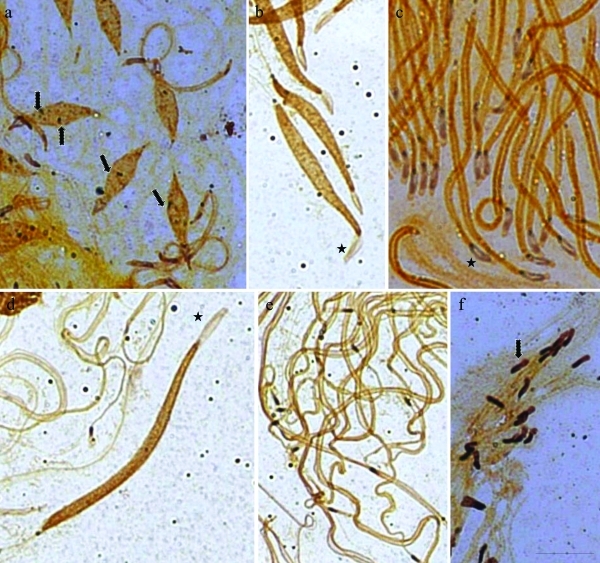
Cells of the seminiferous tubules of *Deois flavopicta* (b, d, e), *Mahanarva fimbriolata* (a, f) and *Notozulia entreriana* (c) stained with silver nitrate: a) Elongating spermatids with some stained areas (dashed arrows); b-e) elongating spermatids with staining in the posterior and anterior region of the head in *D*. *flavopicta* and in *N*. *entreriana*, after the frontal area of the spermatid, which did not show any staining in the head (stars); f) elongating spermatids strongly stained in the entire area of the head (dashed arrow). Scale bar: 10 μm.

The round spermatids showed a round silver stained body (Figure 3l). Silver impregnation was evident at the beginning of spermatid elongation in the medial and posterior regions of the head. (Figure 3m-o). An unstained area could be seen in the frontal, part of the spermatid head, as was also observed after lacto-acetic orcein staining (Figure 3o). Only *M. fimbriolata* spermatids showed silver staining during the beginning of elongation (Figure 4a). In a subsequent stage, the spermatids of *D. flavopicta* and *N*. *entreriana* showed silver impregnation in the posterior and anterior areas. Shortly afterwards, the frontal area of the spermatid (which had no silver staining in the head, Figure 4b-e) and the spermatid of *M*. *fimbriolata* were strongly and uniformly stained in the entire area of the head (Figure 4f).

## Discussion

The cytogenetic analyses of *D. flavopicta*, *M. fimbriolata* and *N. entreriana* allowed the observations that these species have: holocentric chromosomes; kinetic activity restricted to the chromosomes telomeric regions; chiasmata; reductional segregation of the autosomes and equational segregation of the sex chromosome in the first meiotic division; an opposite behavior in the second meiotic division; and late migration of the sex chromosomes during anaphase I. It was possible to observe, therefore, that the species of Auchenorrhyncha investigated displayed cytogenetic characteristics similar to those extensively reported in Heteroptera (Schrader, 1935, 1940; Hughes-Schrader and Schrader, 1961; Buck, 1967; Comings and Okada, 1972; Motzko and Ruthman, 1984; John and King, 1985; Rufas and Giménez-Martín, 1986; Jones, 1987; Solari and Agopian, 1987; González-Garcia *et al.*, 1996; Wolf, 1996; Pérez *et al.*, 1997).

The chromosome complements found in the species analyzed here were 2n = 19 (18A + X0) in *D. flavopicta* and *M.fimbriolata* and 2n = 15 (14A + X0) in *N*. *entreriana*. The three species showed the same X0 sex-determination system. The 2n = 19 karyotype observed in *M.fimbriolata* confirmed the data obtained by Marin-Morales *et al.* (2002). Due to the small number of Auchenorrhyncha species analyzed, it is not possible to establish the exact modal number of chromosomes for this suborder. Because Auchenorrhyncha show holocentric chromosomes, it is hard to establish if the ancestral species had higher or lower chromosome numbers, since both types of chromosome rearrangements, fusion and/or fragmentation, can occur in the species of this group. The definition of the ancestral species chromosome number will depend on further analyses, such as molecular ones.

The three species showed the same results after silver nitrate staining, making it impossible to define exactly in which chromosome the nucleolar organizing region (NOR) was located. Nevertheless, there was an indication that it may be in the terminal and subterminal areas of some autosomes in *D. flavopicta* and *M.fimbriolata*. In *D.flavopicta* silver staining was also seen around the periphery of the autosome. According to other studies on NORs location, it could be in the telomeric region of the X chromosome (*Aphis pomi,* green apple Aphid) (Criniti *et al.*, 2005).

The lack of NORs detection with conventional cytogenetic techniques may be due to the extremely high compaction level of the chromatin during metaphase, which may hinder the contact between the silver ions and the protein acidic residues. It may also result from differences and variability in nucleolar morphologies among species, as already observed, for instance, in mitotic metaphases of plants and in meiotic testicular cells of Heteroptera.

In plants, when the nucleolar bodies are no longer visible, a group of proteins remains associated with the NORs; another group is located in the periphery of the chromosome, where it stays from late prophase until initial telophase; and a third group of proteins and RNAs are evenly distributed in the cytoplasm between prophase and telophase (Ochs *et al.*, 1985; Fakan and Hernandez-Verdun, 1986; Fischer *et al.*, 1991; Wachtler and Stahl, 1993; Schwarzacher and Wachtler, 1993; González-Garcia and Rufas, 1995; Dundr *et al.*, 1997). The meiotic metaphase cells of Heteroptera showed some silver-stained material in the region of the NORs (Rebagliati *et al.*, 2003; Castanhole *et al.*, 2008), on the periphery of the chromosome (Souza *et al.*, 2007a, 2007b), nucleolar semi-persistence (Cattani and Papeschi, 2004), or total disorganization (Risueño and Medina, 1986). Only a small number of species of Auchenorrhyncha have been described, which may be why the only behavior that has been observed was silver stained material in the area of the NOR and on the periphery of the chromosome. The analyses of additional species could reveal total disorganization during metaphase or nucleolar semi-persistence.

Data related to the nucleolar behavior during spermiogenesis are extremely rare in Auchenorrhyncha, but in Heteroptera it was observed that elongating spermatids are silver stained in the posterior region of the head (Pentatomidae, Lygaeidae) (Souza *et al.*, 2007a, 2007b). The distribution pattern of nucleolar proteins during spermatid elongation was the same in *D. flavopicta*, *N. entreriana* and *M. fimbriolata*. This pattern was also similar to that described for some species of Heteroptera.

Auchenorrhyncha and Heteroptera share many characteristics that should be taken into account in evolutionary studies and other techniques should be used to verify the level of similarity among species of these groups.
